# Efficacy and Safety of Non-Anesthesiologist Administration of Propofol Sedation in Endoscopic Ultrasound: A Propensity Score Analysis

**DOI:** 10.3390/diagnostics10100791

**Published:** 2020-10-06

**Authors:** Antonio Facciorusso, Antonio Turco, Carlo Barnabà, Grazia Longo, Graziano Dipasquale, Nicola Muscatiello

**Affiliations:** Gastroenterology Unit, Department of Medical Sciences, Ospedali Riuniti di Foggia, Viale Pinto 1, 71100 Foggia, Italy; antonio883at@libero.it (A.T.); jacopo.fiesco84@gmail.com (C.B.); longomariagrazia70@gmail.com (G.L.); gradipas@gmail.com (G.D.); nicomuscatiello@gmail.com (N.M.)

**Keywords:** EUS, FNA, pancreas, endoscopy, anesthesia

## Abstract

In spite of promising preliminary results, evidence supporting the use of non-anesthesiologist-administered propofol sedation (NAAP) in endoscopic ultrasound (EUS) procedures is still limited. The aim of this manuscript was to examine the safety and efficacy of NAAP as compared to anesthesiologist-administered propofol sedation in EUS procedures performed in a referral center. Out of 832 patients referred to our center between 2016 and 2019, after propensity score matching two groups were compared: 305 treated with NAAP and 305 controls who underwent anesthesiologist-administered propofol sedation. The primary outcome was the rate of major complications. The median age was 67 years and the proportion of patients with comorbidities was 31.8% in both groups. One patient in each group (0.3%) experienced a major complication, whereas minor complications were observed in 13 patients in the NAAP group (4.2%) and 10 patients in the control group (3.2%; *p* = 0.52). Overall pain during the procedure was 2.3 ± 1 in group 1 and 1.8 ± 1 in group 2 (*p* = 0.67), whereas pain/discomfort upon awakening was rated as 1 ± 0.5 in both groups (*p* = 0.72). NAAP is safe and effective even in advanced EUS procedures. Further randomized-controlled trials (RCTs) are warranted to confirm these findings.

## 1. Introduction

Propofol represents a valuable sedative agent in advanced endoscopic procedures. Its use has increased in recent years, due to its favorable pharmacokinetic profile compared with traditional sedative regimens, based on the combination of benzodiazepines and opioids [1,2].

While propofol is generally administered by anesthesia specialists, a growing body of evidence seems to suggest that endoscopists can administer or supervise the nurse-administration of propofol sedation safely without the involvement of a specialist in anesthesia [3].

Non-anesthesiologist-administered propofol sedation (NAAP) has been found, in several studies, to be associated with shorter sedation and recovery times as compared to standard moderate sedation with opioid and benzodiazepine [3,4], thus, supporting its use in routine sedation regimens.

It is well-known that the administration of propofol by anesthesia specialists increases significantly the costs of endoscopic procedures without a consistent and recognized improvement in safety and efficacy outcomes [1,5]. On the other hand, anesthesiology societies claim that NAAP is unsafe, mainly based on the warning contained in the package insert that propofol should be given only by persons trained in the administration of general anesthesia [6,7]. The U.S. Food and Drug Administration recently denied a petition by gastroenterologists seeking the removal of this particular restriction [8]. However, these warnings do not take into account recent evidence on the safety of NAAP in a variety of routine and advanced endoscopic procedures, including endoscopic ultrasound (EUS) [9].

Therefore, although current American Society of Gastrointestinal Endoscopy (ASGE) guidelines recommend the use of NAAP in the presence of adequate specialized training, patient selection, and personnel dedicated to continuous physiologic monitoring [1], regulations regarding administration of propofol are still highly variable and determined at the state, regional, and local levels, regardless of the targeted level of sedation. As a result, the practice of NAAP is quite limited in several countries, including Italy [10].

EUS represents the gold-standard diagnostic tool for the assessment of several gastrointestinal and pancreaticobiliary diseases, mainly because of its ability to allow the performance of EUS-guided fine needle aspiration (FNA) or fine-needle biopsy (FNB) [11,12,13,14]. Furthermore, a number of advanced EUS-guided procedures are increasingly performed in the clinical practice, such as EUS-guided drainage [15] or tumor ablation [16,17].

While routine diagnostic or staging EUS carries a relatively low risk of complications, it is usually more time-consuming and more uncomfortable than a simple diagnostic upper GI tract endoscopy. Moreover, EUS-guided tissue acquisition and advanced EUS procedures are more difficult and lengthier, therefore, a deeper sedation is necessary to fulfil with EUS quality requirements [18].

Although preliminary reports seem to support the use of NAAP, also during EUS-FNA [19], further research is needed to confirm these results and to prove their applicability to other EUS-guided procedures.

The aim of this study was to examine the outcomes of patients undergoing EUS procedures in our center and to compare the safety and efficacy results obtained with endoscopist-administered propofol sedation as compared to propofol sedation in the presence of an anesthesia specialist.

## 2. Materials and Methods

### 2.1. Patients

From a prospectively collected database of 1026 patients referred to our center between January 2016 and December 2019, to be evaluated by means of EUS, data regarding 832 consecutive patients who underwent diagnostic or interventional EUS procedures were reviewed. The Institutional Review Board of the Hospital of Foggia (Azienda Ospedaliera Ospedali Riuniti) approbation for this retrospective report was obtained (n. 4187/2020 approved on 24 June 2020).

Indications to EUS were: (1) uncertain abdominal lesion characterization in radiological imaging studies requiring EUS-guided tissue sampling; (2) pancreatic pseudocysts or peri-pancreatic fluid collections requiring EUS-guided drainage; (3) advanced pancreatic cancer patients with untreatable cancer-related pain referred to be treated with EUS-guided celiac plexus neurolysis (CPN). Exclusion criteria were age <18 years, anesthesiology (ASA) score >III, refusal to sign informed consent, and incomplete data.

Patients in antithrombotic treatment suspended the anticoagulant/antiaggregant agent and underwent bridging therapy with enoxaparin when necessary. No intravenous prophylactic antibiotics before EUS-guided tissue sampling or EUS-CPN were administered as described elsewhere [20,21].

All procedures were performed by board-certified, 20 year-experienced gastroenterologists (NM).

In the first part of the recruitment period, an on-room anesthesiologist was available, and all of the patients underwent anesthesiologist-administered propofol sedation; later on, due to restriction policies at our Institution, sedation was administered only by an endoscopist not directly involved in the procedure (A.F.) or by registered nurses (A.T., C.B., G.L., G.D.).

All of the personnel administering propofol completed a theoretical and practical course with full-scale simulation training in administration and the handling of adverse events. Therefore, both the supporting personnel and the endoscopist performing the procedure had full training in basic and advanced life support. Training implied a 3-day introductory course combining theory and practice with a focus on practical training. Next, clinical training consisted of a learning phase of at least 2 weeks with a mentor and included individual competency assessments. These competency assessments included advanced life support skills (i.e., airway management, defibrillation, and the use of resuscitative medications), training in uninterrupted monitoring of the patient’s clinical and physiologic parameters throughout the procedure including pulse oximetry, electrocardiography, and intermittent blood pressure measurement; ability to rescue patients who become unresponsive or unable to protect their airway or who lose spontaneous respiratory or cardiovascular function.

After exclusion of subjects not fulfilling the inclusion criteria, the study population included two groups of patients: 395 patients undergoing EUS under NAAP (Group 1) and 437 controls (Group 2) who underwent EUS with sedation administered by an anesthesia specialist.

### 2.2. Sedation

The technique used for diagnostic and interventional EUS at our center has been described elsewhere [16,17,20,22,23].

Propofol was administered as intermittent bolus monotherapy, using the same guidelines as for standard endoscopies [24]. Sufficient sedation for easy introduction of the endoscope was achieved with an initial bolus of propofol (0.5–1 mg/kg) administered intravenously, followed by a repeated bolus (10–20 mg), according to the patient’s condition. Maintenance of sedation was achieved with intermittent doses of 10–20 mg if the patient showed signs of discomfort. The dedicated nurse continuously monitored the patient’s vital parameters with pulse oximetry, blood pressure (BP) taken every five minutes, and electrocardiography. Saline infusion (500 mL/h) and supplemental oxygen (3 L/min) flow on a nasal cannula were administered to all patients and initiated a minimum of 3 min prior to sedation.

Depending on complexity of the procedure and comorbidities, patients were either hospitalized for observation for 24 h or had the procedure in day hospital. In both cases, the monitoring protocol was the same.

### 2.3. Outcomes

Data were collected by a research nurse, unblinded to the sedation regimen administered. Adverse event (AE) rates were evaluated during the procedure and before discharge.

Major complications included endotracheal intubation, hospitalization, permanent neurologic impairment, or death [25]. Minor complications included the following: a decline in SpO2 <90% for at least 5 s; a heart rate or blood pressure change by ≥25% from baseline, supplemental O_2_ above the standard 4 L/min, severe coughing that interfered with procedure completion, or restraint of the patient (beyond holding of the hands) longer than 3 s [25]. Patient/endoscopist satisfaction with the sedation and quality of the procedure were rated according to a numeric scale, where 0 was “poor” and 10 “excellent”.

The primary outcome was a major complications rate. Secondary outcomes were minor complications (along with overall complications), patient/endoscopist satisfaction, quality of EUS, overall pain during the procedure, and pain or discomfort upon awakening. Mean propofol dose and procedure duration were also registered.

### 2.4. Statistical Analysis

Categorical variables were reported as number of cases and percentage, and differences between groups were compared using the Chi-square and the McNemar analysis before and after matching, respectively.

Continuous variables were expressed as mean and standard deviation and differences between groups were explored by the Mann–Whitney and Wilcoxon signed-rank test before and after matching, respectively. All analyses were 2-tailed and the threshold of significance was assessed at ≤0.05.

To overcome biases owing to the different distribution of covariates, a 1-to-1 match was created using propensity score analysis. The propensity score represents the probability of each individual patient being assigned to a particular condition in a study given a set of known covariates [26].

The propensity score model was built upon a multivariate logistic regression analysis to predict the probability of each individual patient being submitted to one of the two sedation regimens based on several demographic and clinical covariates, namely age, gender, comorbidities, American Society of anesthesiology (ASA) score (ASA ≤II versus ASA III); type of procedure (EUS-FNA/FNB versus other); number of previous endoscopies; location of the procedure (pancreas versus other).

The predictive values were then used to obtain a 1-to-1 match by using the nearest neighbor matching within a specified caliper distance. Nearest neighbor matching within a specified caliper distance selects for matching an untreated subject whose propensity score is closest to that of the treated subject (“nearest neighbor matching” approach) with the further restriction that the absolute difference in the propensity scores of matched subjects must be below some pre-specified threshold (the caliper distance) [26,27]. Thus, there were patients for whom the propensity score could not be matched because of a greater caliper distance and these subjects were excluded from further analysis. As suggested by Austin, a caliper of width equal to 0.2 of the standard deviation of the logit of the propensity score was used, as this value has been found to minimize the mean squared error of the estimated treatment effect [27,28].

The statistical analysis was performed using the MatchIt package in R Statistical Software 3.0.2 (Foundation for Statistical Computing, Vienna, Austria).

## 3. Results

### 3.1. Patients

The baseline characteristics of the whole series of 832 patients who underwent EUS procedures at our center are reported in Table 1.

Group 1, namely patients treated with NAAP, included 395 patients, whereas 437 underwent EUS under propofol sedation administered by an anesthesia specialist (Group 2). As reported in Table 1, the number of subjects ASA III was significantly superior in group 1 (91 versus 76 patients, respectively; *p* = 0.05). No difference in other baseline parameters was registered between the two groups.

**Figure 1:**
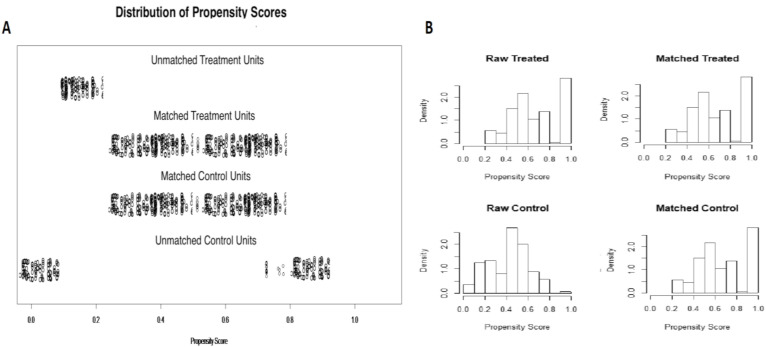
Propensity score matching. Out of the initial 832 patients, after 1-to-1 propensity score caliper matching, 610 patients were selected for comparison: 305 patients treated with non-anesthetist-administered propofol sedation and 305 controls (with anesthesia specialist-administered propofol sedation). (**A**) Propensity score matching jitter plot; (**B**) propensity score matching histogram.

After 1-to-1 propensity score match, 610 patients were selected for comparison: 305 subjects treated with NAAP (Group 1) and 305 controls (Group 2). Details of propensity score matching are shown in Figure 1A,B.

The characteristics of these propensity score-matched patients are reported in Table 2.

Median age was 67 years in group 1 and 66 in group 2 (*p* = 0.91). Most patients were male (60% in both groups; *p* = 1.0) and the proportion of patients with comorbidities was the same in the two groups (31.8%; *p* = 1.0). 

After propensity-score matching, no difference was observed concerning the number of patients ASA III (53 in group 1 and 55 in group 2; *p* = 0.89). The vast majority of patients underwent EUS-tissue acquisition of pancreatic lesions, while the mean number of previous endoscopies was 1.2 and 1.3 in the two groups, respectively (*p* = 0.85).

### 3.2. Safety and Efficacy

A detailed list of the study outcomes is reported in Table 3.

One patient in each group (0.3%) experienced a major sedation-related complication, in particular, one patient in the NAAP group required endotracheal intubation and hospitalization in the intensive care unit for two days due to persistent decline in SpO2, and one patient in the control group required hospitalization because of persistent and significant blood pressure increase (grade 2 hypertension). This patient was monitored for 24 h in an internal medicine ward, but no pharmacological treatment was required. No fatal event was registered in the study.

**Figure 2:**
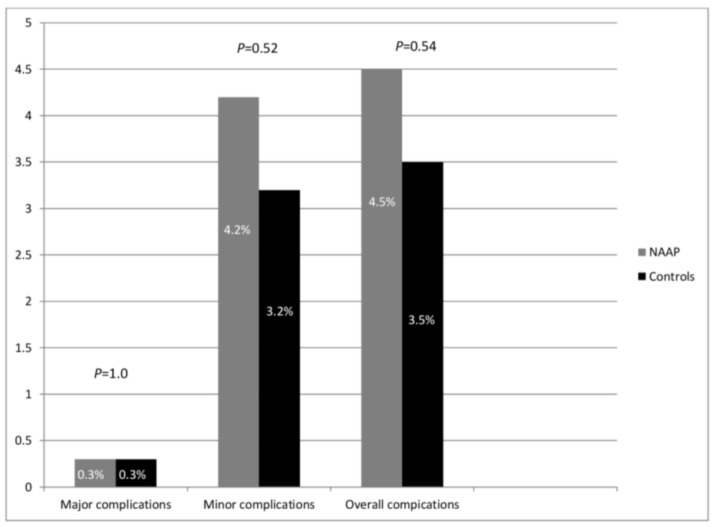
Comparison of major complication, minor complication, and overall complication rates between the two study groups. NAAP, Non-anesthesiologist administered propofol sedation.

Minor complications were observed in 13 patients in the NAAP group (4.2%) and 10 patients in the control group (3.2%; *p* = 0.52, Table 3 and Figure 2). In particular, a transient decline in SpO2 <90% was registered in 10 patients in both groups, whereas 3 NAAP patients required administration of supplemental O_2_ above standard 4 L/min. Patients recovered with no need of pharmacological treatment and they were discharged within 2 h. Mean propofol dose used in the two groups was 85.4 mg and 92.4 mg, respectively (*p* = 0.24). 

As reported in Table 3, no difference in any of the efficacy outcomes was observed between the two groups. Mean patient and endoscopist satisfaction with the procedure were 8.5 and 8.3, respectively, with no difference between the two groups (*p* = 0.88 and *p* = 0.75). Mean quality of the procedure (rated by the endoscopist) was 9 ± 1 and 9 ± 0.8 (*p* = 1.0). Overall pain during the procedure was 2.3 ± 1 in group 1 and 1.8 ± 1 in group 2 (*p* = 0.67), whereas pain/discomfort upon awakening was rated as 1 ± 0.5 in both groups (*p* = 0.72).

Mean procedure duration was similar (20 and 23 min in the two groups, respectively; *p* = 0.62).

## 4. Discussion

Advanced endoscopic procedures usually require adequate sedation in order to decrease patient anxiety, discomfort, and pain; thus, improving patient acceptance and satisfaction. Sedation is also important to medical practitioners as it improves the quality of endoscopic examinations and treatment outcomes [18].

Complex and lengthy procedures, such as diagnostic and interventional EUS, usually require deep sedation. Current guidelines support the use of propofol-based sedation in these procedures as it is able to offer higher patient and endoscopist satisfaction in comparison to opioids plus benzodiazepines, decreasing procedure-related time, as well as recovery time, without increasing the rate of adverse events [1]. On the other hand, deep sedation requires qualified medical providers; due to limited anesthesiology resources in most countries, NAAP sedation has been increasingly used with promising results [29].

Although propofol has a favorable safety and efficacy profile in comparison to other sedative regimens [30,31], the lack of an antidote for reversibility and the potential severe adverse events, including cardiopulmonary compromise requiring resuscitation, call for a particular caution in its use. Therefore, several anesthesiology societies and regulatory agencies recommended against the use of propofol in absence of an anesthesia specialist [6,7,8].

Experienced endoscopists and registered nurses have responded to this demand through implementation of educational programs, definition of clinical competencies, and promulgation of recommended practice guidelines by professional practice organizations and nursing position statements [32].

NAAP is uncommonly used in Italy [10]. Since the Italian Professional Ethics Code (Article 13) [33] recently stated that a physician can prescribe any drug, provided he has adequate knowledge of the drug, and the risks are proportionate to the expected benefits, and since, according to the Italian Drug Agency (Agenzia Italiana del Farmaco, AIFA), propofol usage is limited to hospital settings, but prescription and administration by a specialist is not warranted, the Italian Society of Endoscopy (SIED) proposed to license the administration of propofol by trained personnel not involved in the endoscopic procedure in ASA ≤II and selected ASA III patients [10].

However, still limited evidence supports the use of NAAP in endoscopic ultrasound procedures, hence, the need of further large-series reporting results of NAAP in diagnostic and interventional EUS setting.

In order to overcome the potential biases related to the retrospective nature of the study and to take into account, properly, all confounding variables, we performed a propensity score matching analysis on the basis of several demographic and procedural covariates, thus, two perfectly balanced treatment groups were obtained (Table 2).

Our results confirm that the incidence of major sedation-related complications is very low (0.3%) both in NAAP and anesthesia specialist-administered propofol sedation, a finding in keeping with the current literature [3,24]. Of note, no fatal event was registered in our series.

Likewise, the rate of minor complications was below 5% without difference between the two groups (*p* = 0.52). Episodes of transient decline in SpO2 are commonly reported in the endoscopic practice and they usually do not represent a relevant clinical issue.

Furthermore, NAAP did not lead to an increase in propofol consumption, as the mean propofol dose was slightly lower in NAAP group (85.4 mg versus 92.4 mg, *p* = 0.24). This finding is also in touch with previous reports [25].

All of the quality and efficacy outcomes were comparable between the two groups; thus, confirming the applicability of propofol sedation even in absence of an anesthesia specialist. As a consequence, the mean procedure duration was similar in the two groups (20 and 23 min, respectively; *p* = 0.62).

Therefore, our results confirm that propofol sedation can be administered by adequately trained endoscopists or nurses, even in absence of an anesthesiologist. These findings might be of particular interest in low-resource settings with no availability of an on-room anesthesia specialist.

This study has a number of strengths: first, it is the largest published series directly comparing the safety and efficacy of NAAP to anesthesia specialist-administered propofol sedation in EUS procedures. Secondly, the accurate statistical design and the completeness of the collected data strengthen the results of our analysis. Thirdly, the unicentricity of the current study is a guarantee against eventual biases due to different treatment procedures or endoscopic trainings.

Nevertheless, the paper has some weaknesses. Its main limitation is the retrospective nature of the study, which could have led to selection biases. However, a propensity score matching analysis based on the baseline covariates known to influence post-procedural outcomes was performed in order to obviate to the aforementioned bias. In addition, the overall low rate of major complications did not enable us to perform a specific subgroup analysis based on baseline parameters. Finally, cost-effectiveness analysis was beyond the scope of the current study.

## 5. Conclusions

Despite such limitations, our analysis provides robust evidence supporting the favorable efficacy and safety profile of NAAP in EUS procedures. Our results should push an extensive use of NAAP, even in advanced endoscopic ultrasound and in low-resource settings where the persistent presence of an anesthesia specialist is not available. Further series and randomized-controlled trials (RCTs) are warranted to confirm these findings.

## Figures and Tables

**Table 1:** Baseline patients’ characteristics before propensity score matching.

Variable	NAAP (*n* = 395)	Controls (*n* = 437)	*p* Value
Age (years)	68 ± 9	65 ± 7	0.54
Gender: Male	229 (57.9%)	279 (63.8%)	0.08
Female	166 (42.1%)	158(36.2%)	
Comorbidities	128 (32.4%)	143 (32.7%)	0.77
ASA score ≤II	304 (76.9%)	361 (82.6%)	0.05
EUS-FNA/FNB	315 (79.7%)	345 (78.9%)	0.77
Other EUS procedures	80 (20.3%)	92 (21.1%)	
Number of previous endoscopies	1.3 ± 1.2	2.1 ± 1.5	0.65
Location: Pancreas	305 (77.2%)	352 (80.5%)	0.26
Other	90 (22.8%)	85 (19.5%)	

Continuous variables were reported as mean values and standard deviations. Comparisons were performed with Mann–Whitney *U* test for continuous variables and Fisher exact test for categorical ones. Abbreviations: ASA, American Society of Anesthesiology; EUS, endoscopic ultrasound; FNA, fine-needle aspiration; FNB, fine-needle biopsy; NAAP, non-anesthesiologist-administered propofol sedation. Significances are reported in bold.

**Table 2:** Baseline patients’ characteristics after propensity score matching.

Variable	NAAP (*n* = 305)	Controls (*n* = 305)	*p* Value
Age (years)	67 ± 3	66 ± 9	0.91
Gender: Male	183 (60%)	183 (60%)	1.0
Female	122 (40%)	122 (40%)	
Comorbidities	97 (31.8%)	97 (31.8%)	1.0
ASA score ≤2	252 (82.6%)	250 (81.9%)	0.89
EUS-FNA/FNB	248 (81.3%)	250 (81.9%)	0.93
Other EUS procedures	57 (18.7%)	55 (18.1%)	
Number of previous endoscopies	1.2 ± 1	1.3 ± 1.3	0.85
Location: Pancreas	250 (81.9%)	248 (81.3%)	0.93
Other	55 (18.1%)	57 (18.7%)	

Continuous variables were reported as mean values and standard deviations. Comparisons were performed with Mann–Whitney *U* test for continuous variables and Fisher’s exact test for categorical ones. The following variables were selected for propensity score calculation: age, gender, comorbidities, ASA score; type of procedure; number of previous endoscopies; location of the procedure. Abbreviations: ASA, American Society of Anesthesiology; EUS, endoscopic ultrasound; FNA, fine-needle aspiration; FNB, fine-needle biopsy; NAAP, non-anesthesiologist-administered propofol sedation. Significances are reported in bold.

**Table 3:** Study outcomes.

	NAAP (305 Patients)	Controls (305 Patients)	*p*-Value ^a^
***Safety***			
• Any major complications *	1 (0.3%)	1 (0.3%)	1.0
**• Any minor complications ^+^**	13 (4.2%)	10 (3.2%)	0.52
**• Mean propofol dose (mg)**	85.4 ± 25.5	92.4 ± 30.5	0.24
***Efficacy***			
**• Patient satisfaction with the procedure ^§^**	8.5 ± 1.5	8.3 ± 1.5	0.88
**• Endoscopist satisfaction with the procedure ^§^**	8.3 ± 2	8.2 ± 1.8	0.75
**• Quality of EUS ^§^**	9 ± 1	9 ± 0.8	1.0
**• Overall pain during the procedure ^§^**	2.3 ± 1	1.8 ± 1	0.67
**• Pain or discomfort upon awakening ^§^**	1 ± 0.5	1 ± 0.5	0.72
**• Mean procedure duration (min)**	20 ± 23	23 ± 18	0.62

Variables are expressed as absolute number (percentage). ^a^ Calculated by means of McNemar test. * Endotracheal intubation, hospitalization, permanent neurologic impairment, or death. ^+^ Decline in SpO2 <90%; heart rate or blood pressure change by ≥25% from baseline; administration of supplemental O2 above standard 4 L/min; severe coughing that interfered with completion of the procedure. ^§^ Poor is 0, excellent is 10. Abbreviations: EUS, endoscopic ultrasound; NAAP, non-anesthesiologist-administered propofol sedation.
